# Assessing the Short-Term Efficacy and Safety of Guselkumab for Moderate-to-Severe Plaque Psoriasis: Meta-Analysis of Randomized Controlled Trials

**DOI:** 10.1155/2020/4975628

**Published:** 2020-07-17

**Authors:** Jing Yang, Zongming Wang, Xilin Zhang

**Affiliations:** Department of Dermatology and Venereology, Chong Qing Three Gorges Central Hospital, Chongqing 404100, China

## Abstract

**Background:**

To investigate the efficacy and safety of guselkumab in the treatment of moderate-to-severe plaque psoriasis.

**Methods:**

A systematic review was undertaken to identify double-blind randomized controlled trials (RCTs). PubMed, Web of Science, Cochrane Library, EMBASE, and Google Scholar databases were searched before 1 March 2020. The odds ratios (ORs) with 95% confidence interval (CI) were calculated. All analyses were conducted with intention-to-treat basis. A range of sensitivity analyses was undertaken.

**Results:**

A total of 7 articles contained 1206 plaque psoriasis patients with guselkumab, 585 patients with placebo, and 1250 patients with adalimumab were included. The results indicated that guselkumab had better efficacy than placebo or adalimumab for Psoriasis Area and Severity Index score reductions from baseline of 75% (PASI 75) (OR = 61.37, 95% CI = 31.15 to 120.91; OR = 3.08, 95% CI = 2.35 to 4.06), Investigator's Global Assessment scores of 0 or 1 (IGA 0/1) (OR = 65.75, 95% CI = 45.54 to 94.95; OR = 2.79, 95% CI = 2.17 to 3.59), and Dermatology Life Quality Index scores of 0 or 1 (DLQI 0/1) (OR = 29.64, 95% CI = 18.80 to 46.73; OR = 1.86, 95% CI = 1.50 to 2.31). The guselkumab had similar safety with placebo or adalimumab about the incidence of adverse events (AEs) (OR = 1.05, 95% CI = 0.86 to 1.29; OR = 0.97, 95% CI = 0.79 to 1.19) and serious adverse events (SAEs) (OR = 1.03, 95% *C*I = 0.47 to 2.27; OR = 0.91, 95% CI = 0.44 to 1.87). Meanwhile, there was no statistically significant association of infections and serious infections compared with the placebo or adalimumab group. The guselkumab was more effective and had the similar tolerance.

**Conclusion:**

The guselkumab had excellent efficacy and great safety in moderate-to-severe plaque psoriasis, but long-term safety remained to be determined.

## 1. Introduction

Psoriasis is a chronic immune-mediated inflammatory skin disease that manifests in the skin or joint [1]. It affects nearly 1~3% of the population worldwide and has brought enormous financial pressure to the patients [2]. The etiology of psoriasis is unknown; it may be associated with the defect in proliferation and differentiation of keratinocytes and inflammatory cell infiltration. The subtypes of psoriasis are diverse; 90% of the total number of patients are plaque psoriasis [3, 4]. The lesions of plaque psoriasis show clear red plaques, which are covered with silvery white scales and accompanied by obviously itching symptoms. The main characteristics of plaque psoriasis are infiltrative plaques and thicker scales with light red to dark red. If the scales are scraped out, a thin film phenomenon and spotted bleeding could be seen. They usually have a longer course [5].

At present, the main inhibitors for the treatment of psoriasis are IL-23 inhibitors, IL-17 inhibitors, and TNF-*α* inhibitors. The central role of interleukin-23/interleukin-17 (IL-23/IL-17) axis in the pathogenesis of psoriasis and the effectiveness of its targeted therapy have been confirmed by numerous studies [6, 7]. IL-23 belongs to the IL-12 cytokine family. It is a heterodimer composed of p40 and p19 subunits [8]. Guselkumab is a fully human immunoglobulin G 1*λ* (IgG 1*λ*) monoclonal antibody that blocks the downstream signaling of IL-23 by specifically binding to the p19 subunit of IL-23 [9]. As a proinflammatory factor, TNF-*α* is produced by a variety of skin immune cells and could regulate the production of IL-23. At the same time, they cooperate with IL-17 to promote keratinocytes to express various psoriasis-related inflammatory factors. Therefore, TNF-*α* inhibitors have shown remarkable effects in the treatment of plaque psoriasis. Adalimumab is the first successfully developed fully human IgG, which has a high affinity for soluble TNF-*α*, and could effectively counteract the biological function of TNF-*α* by blocking the interaction between TNF-*α* and its receptors P55 and P75. Thus, the condition of psoriasis patients has been improved [10]. Currently, the guselkumab was in the phase III clinical trials for the treatment of moderate-to-severe plaque psoriasis and the phase II clinical trials for the treatment of arthritis psoriasis. The adalimumab was in the phase III clinical trial for the treatment of psoriasis. Relevant clinical trials of guselkumab showed that the Psoriasis Area and Severity Index (PASI) scores were decreased significantly after treatment and showed good safety [11–13]. Kim et al. [14] indicated that adalimumab treatment for moderate to severe plaque psoriasis was associated with greater PASI reduction, higher rates of resolution of skin signs and symptoms, and greater improvements in dermatological life quality. The studies showed that the effects of anti-IL-23p19 inhibitors were better than those of the IL-17A inhibitors, and they had a shorter induction period and a lower loading dose [15].

Many studies have proved that guselkumab was effective and safe, but some results showed inconsistent conclusions. Gordon et al. [16]. indicated that the infection rate of guselkumab was higher than that of placebo or adalimumab, which was different from other studies. Additionally, there was no study or analysis comparing the efficacy or safety of guselkumab with placebo or adalimumab. This meta-analysis is the first comprehensive analysis of the efficacy and safety of guselkumab, so as to provide further reliable basis for clinical application.

## 2. Materials and Methods

### 2.1. Study Identification

**Figure 1:**
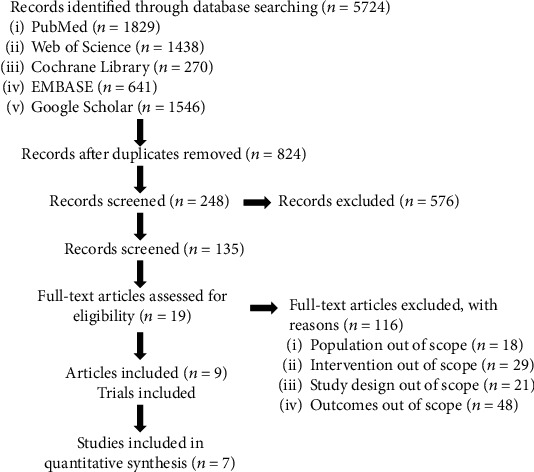
Flowchart of study selection.

The electronic databases including PubMed, Web of Science, Cochrane Library, EMBASE, and Google Scholar databases were searched from 1 January 2000 to 1 January 2020 for studies published in English. The double-blind randomized controlled trials (RCTs) investigating the efficacy and safety of guselkumab were systematically retrieved. Keywords and search strategy were as follows: “IL-23 inhibitor” or “IL-23” or “IL-23p19” or “anti-IL-23” or “guselkumab” or “CNTO1959” combined with “psoriasis.” Comments, editorials, and letters were removed. In addition, the references of these articles were also screened to find other relevant articles. The search strategy is shown in Figure 1.

### 2.2. Study Selection

Trials were selected based on the following inclusion criteria: (1) the study design was limited to double-blind, randomized, placebo-controlled trials; (2) the patients were all older than 18 years, and they had stable (≥6 months) moderate-to-severe chronic plaque at baseline with Body Surface Area (BSA) involvement of 10% or greater; (3) the studies should provide at least one efficacy outcome for short-term treatment: the reduction from baseline in the Psoriasis Area and Severity Index 75 (PASI 75), Investigator's Global Assessment scores of 0 or 1 (IGA 0/1), or Dermatology Life Quality Index scores of 0 or 1 (DLQI 0/1); (4) the studies should provide at least one safety outcome for short-term treatment: one or more adverse events (AEs) and one or more serious adverse events (SAEs); (5) the follow-up time was 16 or 24 weeks. The exclusion criteria were as follows: (1) the patients with psoriasis who were under 18 years of age; (2) the patients of active inflammatory diseases that could have interfered with study assessments who were ineligible, for example, drug-induced psoriasis and guttate, erythrodermic, or pustular psoriasis; (3) the women who were pregnant, breastfeeding, or planning to become pregnant; (4) the patients who had had prior exposure to the study drug or undergone major surgery 12 weeks or less before randomization, and the surgery was planned within 12 months after screening; (5) the patients who had history of allergy or hypersensitivity to a systematically administrated biologic agent; (6) the case-control studies, cohort studies, review articles, conference abstracts, case reports, and unpublished articles.

### 2.3. Data Abstraction and Quality Assessment

Two researchers independently extracted the following information from each study: study design, baseline patient characteristics, interventions, national clinical trial number, IGA, BSA, PASI, and DLQI. The efficacy parameters were PASI 75, PASI 90, PASI 100, IGA 0/1, and DLQI 0/1. The safety parameters were AEs, SAEs, and infections and serious infections. And the PASI 75, IGA 0/1, and DLQI 0/1 were primary indices; the other parameters were secondary indices. The AEs, SAEs, and infections and serious infections were safety indices. The methodological quality of included studies was assessed by one independent reviewer. Any disagreements were discussed with the third researcher.

### 2.4. Statistical Analysis

The efficacy and safety of guselkumab were assessed and compared with a placebo or adalimumab. We performed meta-analysis to calculate odds ratios (ORs) and 95% CIs using the *Mantel-Haenszel* statistical method. A random effects model was used to pool the data, and *I*^2^ statistic was evaluated heterogeneity between summary data. Sensitivity analysis was performed by excluding low-quality studies. All meta-analyses were performed using RevMan version 5.3. All tests were 2-tailed, and *P* < 0.05 was considered statistically significant.

## 3. Results

### 3.1. Literature Search and Study Characteristics

From the searches for studies, 5724 potentially eligible records were identified. Titles and abstracts of these articles were screened for inclusion. Finally, 7 articles that contained 1206 plaque psoriasis patients with guselkumab, 585 patients with placebo, and 1250 patients with adalimumab were included. The process of study selection is shown in Figure 1. The characteristics of enrolled studies are represented detailedly in Table 1.

### 3.2. Risk of Bias

**Figure 2:**
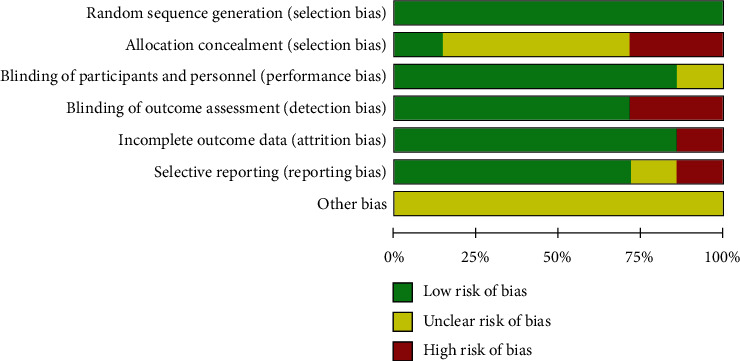
Risk of bias summary.

The methodological quality for the included studies was assessed independently by two researchers based on Cochrane risk-of-bias criteria, and each quality item was graded as low risk, high risk, or unclear risk. The 7 items used to evaluate bias in each trial included (1) random sequence generation, (2) allocation concealment, (3) blinding of participants and personnel, (4) blinding of outcome assessment, (5) incomplete outcome data, (6) selective reporting, and (7) other bias. Overall, the risk of bias for most of the studies was judged to be low (Figure 2).

### 3.3. Primary Outcomes

**Figure 3:**
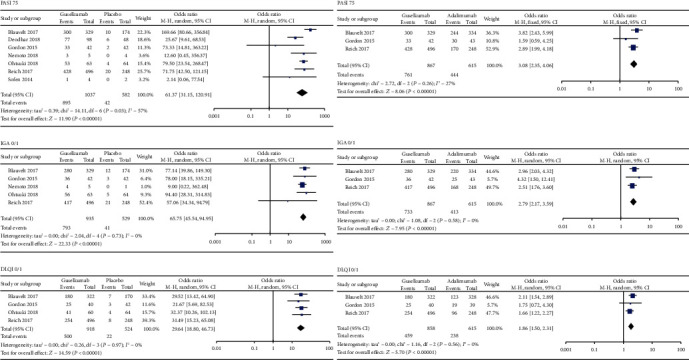
Primary efficacy outcomes of guselkumab in the treatment of plaque psoriasis versus placebo or adalimumab. PASI: Psoriasis Area and Severity Index; IGA: Investigator's Global Assessment; DLQI: Dermatology Life Quality Index.

All tests were conducted using a random effects model. As shown in Figure 3, there were significant differences in PASI 75 (OR = 61.37, 95% CI = 31.15 to 120.91), IGA 0/1 (OR = 65.75, 95% CI = 45.54 to 94.95), and DLQI 0/1 (OR = 29.64, 95% CI = 18.80 to 46.73) between the guselkumab group and the placebo group. There were significant differences in PASI 75 (OR = 3.08, 95% CI = 2.35 to 4.06), IGA 0/1 (OR = 2.79, 95% CI = 2.17 to 3.59), and DLQI 0/1 (OR = 1.86, 95% CI = 1.50 to 2.31) between the guselkumab group and the adalimumab group.

### 3.4. Secondary Outcomes

**Figure 4:**
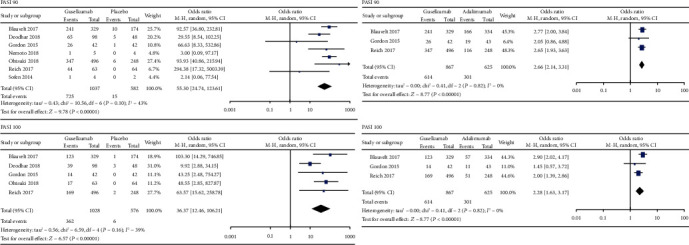
Secondary efficacy outcomes of guselkumab in the treatment of plaque psoriasis versus placebo or adalimumab. PASI: Psoriasis Area and Severity Index.

As shown in Figure 4, there were significant differences in PASI 90 (OR = 55.3, 95% CI = 24.74 to 123.61) and PASI 100 (OR = 36.37, 95% CI = 12.46 to 106.21) between the guselkumab group and the placebo group. Meanwhile, there were significant differences in PASI 90 (OR = 2.66, 95% CI = 2.14 to 3.31) and PASI 100 (OR = 2.28, 95% CI = 1.63 to 3.17) between the guselkumab group and the adalimumab group.

### 3.5. Safety of Guselkumab

**Figure 5:**
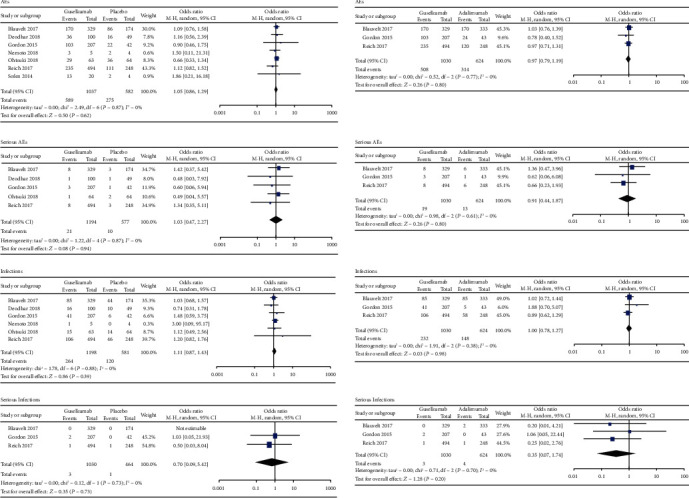
Safety outcomes of guselkumab in the treatment of plaque psoriasis versus placebo or adalimumab. AEs: adverse events; SAEs: serious adverse events.

As shown in Figure 5, guselkumab was well tolerated and the incidence of AEs (OR = 1.05, 95% CI = 0.86 to 1.29) and serious AEs (OR = 1.03, 95% CI = 0.47 to 2.27) were similar to that of the placebo. There was no statistically significant association of infections (OR = 1.11, 95% CI = 0.87 to 1.43) and serious infections (OR = 0.70, 95% CI = 0.09 to 5.42) compared with the placebo group. Compared to the adalimumab group, the incidences of AEs (OR = 0.97, 95% CI = 0.79 to 1.19), serious AEs (OR = 0.91, 95% CI = 0.44 to 1.87), and infections (OR = 1.00, 95% CI = 0.78 to 1.27) and serious infections (OR = 0.35, 95% CI = 0.07 to 1.74) in guselkumab group were not significantly different.

## 4. Discussion

Recently, although TNF inhibitors were been widely used and the traditional treatment strategies of psoriasis were changed, but some refractory patients still might have inhibitor resistance. Meanwhile, studies reported that the intervention of IL-23 in susceptible mice could lead to psoriasis-like lesions, and the expression of IL-23 was elevated in the human psoriasis tissue [17–19], which further testified that IL-23 might be a pathogenic factor of human psoriasis. Blauvelt et al. [19] demonstrated that guselkumab, IL-23p19 inhibitor, was effective in treating plaque psoriasis. In this meta-analysis, the PASI, IGA, and DLQI were used as the main efficacy indicators and AEs and SAEs as the main safety indicators to comprehensively analyze and compare the efficacy and safety of guselkumab.

In this meta-analysis, there was moderate heterogeneity between the enrolled studies (0% < *I*^2^ < 57%); hence, the random effects model was performed. The reason for heterogeneity might be the sample size. The reports by Howard et al. and Nemoto et al. only included several patients, and they did not adjust for the number of participants, which might have increased the probability of smaller *P* values and narrower CIs between the guselkumab and placebo groups. The results in this study showed that there were significant positive benefits for the guselkumab on the PASI 75, PASI 90, PASI 100, IGA 0/1, and DLQI 0/1 compared with placebo or adalimumab. It was consistent with the conclusions of several reviews [20–22]. The incidences of AEs, serious AEs, and infections and serious infections were not significantly different between the groups. The result of infections was inconsistent with some studies. The study of Xu et al. [23] showed that guselkumab might increase the incidence of infections compared with placebo, but there were not existing research reports that the infections had evolved into a serious infection or other SAEs. It was consistent with this meta-analysis. Therefore, the results showed that guselkumab was likely the very efficacious treatment. In previous studies, guselkumab had not been directly investigation, and the results of this study might provide some indirect evidence for clinical application. But the conclusions still needed to be verified by RCTs with a lager sample size. Recent researches by Ohtsuki et al. [24] and Reich et al. [25] showed that guselkumab was effective and safety for the treatment of moderate-to-severe plaque psoriasis. It was to be regretted that the studies were not included, because they had very serious heterogeneous.

Adalimumab is a biological agent targeted at TNF-*α*, which has been proved to have good efficacy in other autoimmune diseases [26]. Although its efficacy in psoriasis was better than placebo, it was unknown compared with guselkumab. The results of this meta-analysis showed guselkumab had superior efficacy to adalimumab in the achievements of PASI 75, PASI 90, PASI 100, IGA 0/1, and DLQI 0/1, but there were no significant differences in incidence rates of safety indicators. They might have similar tolerances.

Sensitivity analyses that excluded low-quality trials and studies that exclusively enrolled patients with particular medical conditions did not alter these results. It was indicated that our results were statistically robust. Publication bias was not reported because the number of trials was less than 10 for each comparison. There were several limitations in this study. First, some comparisons and analyses could not be done, because the RCTs about them have not been done or published. Second, long-term safety needed to be further confirmed by long-term clinical trials. Finally, the quantity and sample size of the literatures were not perfect; the data were deficiency. Accordingly, the efficacy and safety of guselkumab needed to be discussed later.

In this meta-analysis, data were updated compared with prior reports. The subgroup analysis was not performed in this meta-analysis because the included trials were limited. This meta-analysis showed that guselkumab had good efficacy and safety in patients with moderate-to-severe plaque psoriasis and had a better efficacy than adalimumab without other adverse events. But long-term safety and the maintenance of efficacy remained to be determined; future studies should focus more on long-term follow-up.

## Figures and Tables

**Table 1:** Characteristics of RCTs included in this meta-analysis.

Authors (years)	NCT	Medications	Total (*N*)	Age (years)	Male, *N* (%)	Time of follow-up (week)	Duration of psoriasis (year)	BSA (%)	PASI	DLOI
Sofen 2014 [17]	NA	GUS 100 mg	5	NA	4 (80)	16	NA	NA	NA	NA
		Placebo	4	NA	3 (75)		NA	NA	NA	NA
Gordon 2015 [16]	NCT01483599	GUS 100 mg	208	44.0	149 (72)	16	18.5 ± 12.17	24.6 ± 14.48	20.9 ± 8.05	NA
		Ada 40 mg	43	50.0	30 (70)		19.3 ± 12.79	26.8 ± 16.80	20.2 ± 7.58	NA
		Placebo	42	46.5	28 (67)		18.0 ± 13.30	27.5 ± 19.26	21.8 ± 9.98	NA
Reich 2017 [18]	NCT02207244	GUS 100 mg	496	43.7 ± 12.2	349 (70.4)	16	17.9 ± 12.0	28.5 ± 16.4	21.9 ± 8.8	14.7 ± 6.9
		Ada 80 mg	248	43.2 ± 11.9	170 (68.5)		17.6 ± 11.7	29.1 ± 16.7	21.7 ± 9.0	15.0 ± 6.9
		Placebo	248	43.3 ± 12.4	173 (69.8)		17.9 ± 11.9	28.0 ± 16.5	21.5 ± 8.0	15.1 ± 7.2
Blauvelt 2017 [19]	NCT02207231	GUS 100 mg	329	43.90 ± 12.74	240 (72.9)	16	17.90 ± 12.27	28.30 ± 17.10	22.10 ± 9.49	14.00 ± 7.48
		Ada 80 mg	334	42.90 ± 12.58	249 (74.6)		17.00 ± 11.27	28.60 ± 16.66	22.40 ± 8.97	14.40 ± 7.29
		Placebo	174	44.90 ± 12.90	119 (68.4)		17.60 ± 12.44	25.80 ± 15.93	20.40 ± 8.74	13.30 ± 7.12
Nemoto 2018 [11]	NCT01484587	GUS 100 mg	5	NA	4 (80)	16	18.4 ± 13.62	37.8 ± 25.29	19.6 ± 8.51	NA
		Placebo	4	NA	3 (75)		28.8 ± 6.45	22.0 ± 12.96	18.7 ± 4.73	NA
Deodhar 2018 [12]	NCT02319759	GUS 100 mg	100	47.4 ± 12.8	52 (52)	24	7.0 ± 7.2	17.2 ± 15.6	12.0 ± 10.5	NA
		Placebo	49	44.2 ± 12.4	24 (49)		6.9 ± 7.2	13.6 ± 12.5	9.9 ± 8.0	NA
Ohtsuki 2018 [13]	NCT02325219	GUS 100 mg	63	47.8 ± 11.07	47 (74.6)	16	14.39 ± 9.227	37.9 ± 21.48	26.73 ± 12.196	10.3 ± 7.27
		Placebo	64	48.3 ± 10.56	54 (84.4)		13.66 ± 10.291	33.6 ± 18.39	25.92 ± 12.341	10.6 ± 7.74

## Data Availability

This article does not involve any basic experiments and clinical investigations. It only requires a database search, and these data are public and free.
